# Haematometry and Haematocolpos

**Published:** 1914-08

**Authors:** 


**Haematometry and Haematocolpos.** L. Dieulafe of Toulouse, Gaz. des Prat., June 15, 1914, reports a case in a girl of 18. She began to menstruate at 16, without flow of blood, and with painful tumefaction of the abdomen. Following a fall into a ditch, she had a discharge of dark bloody faeces and the tumor disappeared, gradually reforming. The tissues were opened through an H-shaped incision in the vestibule and a large drain inserted after removing the accumulated clots. Recovery uneventful. Subsequent menstruation normal and the patient has married, no longer suffers, and is satisfied with her conjugal relations.

